# The Role of Cancer-Associated Fibroblasts in Cancer Invasion and Metastasis

**DOI:** 10.3390/cancers13184720

**Published:** 2021-09-21

**Authors:** Paris Jabeen Asif, Ciro Longobardi, Michael Hahne, Jan Paul Medema

**Affiliations:** 1 Center for Experimental and Molecular Medicine, Cancer Center Amsterdam, Amsterdam UMC, University of Amsterdam, 1105 AZ Amsterdam, The Netherlands; p.j.asif@amsterdamumc.nl (P.J.A.); c.longobardi@amsterdamumc.nl (C.L.); 2 Oncode Institute, Amsterdam UMC, University of Amsterdam, Meibergdreef 9, 1105 AZ Amsterdam, The Netherlands; 3 Centre National de la Recherche Scientifique (CNRS), Institut de Génétique Moléculaire de Montpellier, Université de Montpellier, 34090 Montpellier, France; michael.hahne@igmm.cnrs.fr

**Keywords:** cancer-associated fibroblast, invasion, metastasis, colorectal cancer, extracellular matrix, epithelial-to-mesenchymal transition, CAF-targeted therapy

## Abstract

**Simple Summary:**

Cancer metastasis is often associated with a dismal prognosis for the patient. It is, therefore, crucial to understand the mechanisms behind the cascade of events that lead to metastatic disease. Cancer-associated fibroblasts (CAFs) play a key role in cancer progression. Therefore, it is of importance to understand the roles of CAFs in invasion, metastasis, and therapy resistance. Here, we reviewed the crosstalk between CAFs and tumor cells to summarize the current knowledge on CAF roles in cancers to provide the necessary structure to advance the field.

**Abstract:**

Cancer-associated fibroblasts (CAFs) play a key role in cancer progression by contributing to extracellular matrix (ECM) deposition and remodeling, extensive crosstalk with cancer cells, epithelial-to-mesenchymal transition (EMT), invasion, metastasis, and therapy resistance. As metastasis is a main reason for cancer-related deaths, it is crucial to understand the role of CAFs in this process. Colorectal cancer (CRC) is a heterogeneous disease and lethality is especially common in a subtype of CRC with high stromal infiltration. A key component of stroma is cancer-associated fibroblasts (CAFs). To provide new perspectives for research on CAFs and CAF-targeted therapeutics, especially in CRC, we discuss the mechanisms, crosstalk, and functions involved in CAF-mediated cancer invasion, metastasis, and protection. This summary can serve as a framework for future studies elucidating these roles of CAFs.

## 1. Introduction

The word “stroma” was derived from Greek and originally meant “covering” or “mattress”, but, in 1835, it entered the field of biology as “a part of a tissue or organ with a structural or connective role”. The stroma consists of many different cell types, but, in this review, we will focus on fibroblasts and their role in cancer invasion and metastasis.

Many articles describe a parallel dialogue between cancer-associated fibroblasts (CAFs) and tumor cells. This crosstalk enables cancer cells to attract fibroblasts and transform them into CAFs, and, in turn, these can modulate and protect cancer cells.

CAFs have been receiving a growing appreciation in the past few years. The impact of CAFs on cancer invasion and metastasis occurs through remodeling of the extracellular matrix (ECM), modulation of epithelial-to-mesenchymal transition (EMT) in cancer cells, secretion of growth factors supporting cancer cells, and influencing therapy responses. It is believed that this is mainly achieved by signaling between CAFs, cancer cells, and ECM exerted either by direct contact, secretion of cytokines, or extracellular vesicles (EVs).

When cancers metastasize, this often is associated with a dismal prognosis for the patient. It is, therefore, crucial to understand the mechanisms behind the cascade of events that lead to metastatic disease. In this cascade, the extracellular matrix (ECM) plays an important role, as it provides structural and biochemical support to cells, but also restrains dispersion of cells. CAFs are known to modify the ECM during tumor progression, making it more permissive for tumor invasion into the surrounding tissue. Key secreted components for ECM remodeling are transforming growth factor beta (TGF-β), hepatocyte growth factor (HGF), and specific interleukins and metalloproteases. These factors aid tumor cells in invasion and metastasis and are interesting targets for therapy, which we will refer to as CAF-targeted therapy.

In this review, we summarize the literature highlighting the recent findings on the role of CAFs in invasion and metastasis to deduce the most promising mechanisms to target, and to find the most urgent remaining knowledge gaps.

## 2. The Role of Fibroblasts in the Initiation of Invasion and Metastasis

**Figure 1:**
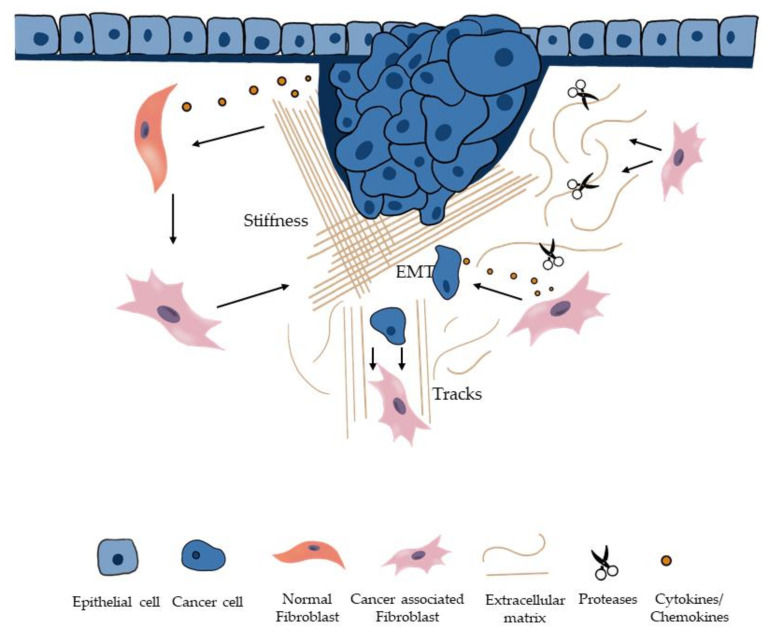
ECM remodeling and EMT modulated by CAFs. CAFs can modify ECM to promote invasion and metastasis of cancer cells by: inducing matrix stiffness; creating tracks for cancer cell invasion; secreting proteases and cytokines. CAFs can also induce EMT.

Plato and Aristotle used the Greek word “metastasis” to describe change by revolution of a political constitution [1]. In modern day society, the word metastasis is mostly related to cancer, with a meaning derived from Greek implying “rapid transition from one point to another”. The metastatic cascade in cancers can be divided into five main processes: invasion, intravasation, circulation, extravasation, and colonization. CAFs can promote ECM remodeling in different ways: by secreting factors, enzymes, and miRNAs; by the generation of ECM tracks; and by inducing matrix stiffness (Figure 1) [2,3]. When we talk about CAFs, we usually refer to a highly heterogeneous population of cells with different functions. One of the ways to explain this heterogeneity could be the different origins that CAFs may have. Although the majority of CAFs appear to originate from tissue resident fibroblasts [4,5], recent research suggests that other origins of CAFs exist. It has been shown that bone-marrow-derived mesenchymal stromal cells could give rise to a subpopulation of CAFs that, in contrast to tissue resident fibroblasts, do not express platelet-derived growth factor receptor A (PDGFRα) [6]. Another source of CAFs could be mature adipocytes [7], and even tumor cells after the process of EMT, which will be discussed later.

In recent years, the efforts of researchers have focused on understanding the differences between the various CAF subpopulations in various tumor types in order to derive clinical benefits. In pancreatic ductal adenocarcinoma (PDAC), two different CAF subtypes were identified with different intratumoral localization and different transcriptomic profiles. Myofibroblastic CAFs (myCAFs) have a direct interaction with neoplastic cells and high expression of alpha-smooth-muscle actin (αSMA), while inflammatory CAFs (iCAFs) are located distally from the tumor cells and secrete high levels of IL-6 and some other cytokines [8]. Similarly, in breast cancer, Friedman et al. described two main subpopulations of CAFs: pCAFs and sCAFs, characterized by the expression of podoplanin (Pdnp) and S100A, respectively. The function and the composition of these CAFs change during tumor progression and the ratio between these populations is an indication of the clinical outcome [9]. In accordance with this, the phenotypes of CAFs change during mammary tumor progression [10]. In CRC, CAF-A and CAF-B were identified as two subtypes with different gene expressions [11]. As CAF subpopulations are being studied more extensively, the field is being updated frequently. These are only a few examples of works which try to shed light on the different CAFs in the TME, and recent reviews are available on the topic [12,13].

One of the big limitations of studying CAFs is the absence of markers that can be used to specifically identify all subpopulations. To overcome this problem, different markers, such as vimentin, αSMA, PDGFRα/β, and fibroblast activation protein α (FAP), are used in combination to identify all the different subpopulations of CAFs in the tumor [14].

In conclusion, further research needs to be conducted in order to define different CAF subpopulations in different tumor types and exploit this heterogeneity for clinical benefits.

### 2.1. CAFs and ECM Remodeling

The ECM is a 3D scaffold that consists of around 300 unique macromolecules that provide mechanical structure and chemical cues for cellular and tissue organization. The ECM also binds factors related to growth, motility, survival, and angiogenesis, such as EGF, TGF-β, HGF, VEGF, and others. The core matrisome encompasses mainly collagens and glycoproteins and can be modified by secreted remodeling enzymes, such as oxidases and proteases [15]. Cells are anchored in this ECM, which provides a stabilizing structure and a framework that influences cell proliferation and survival, but also harbors other physiological and biochemical cues for the cells [16]. When cells receive incoming signals from the tiny ECM protrusions, called filopodia, the actin skeleton can be rearranged [16,17].

ECM modification is a physiological process that mostly occurs during development, tissue regeneration, and wound healing [3,18]. Remodeling mechanisms include deposition, modification, degradation, and organization, which are strictly regulated in normal conditions. Fibroblasts are the main producers of ECM components and ECM-remodeling enzymes that contribute to stromal homeostasis [3]. However, in cancer, these mechanisms are deregulated. Tumor growth and metastasis are highly dependent on the crosstalk between tumor cells and their microenvironment. Some of the main contributors of cancer-related ECM remodeling are deregulated lysyl oxidases (LOXs), matrix metalloproteinases (MMPs), and transglutaminases (TGMs) [2,19,20]. For example, in CRC, MMPs have been shown to significantly change the ECM composition. Under physiological conditions MMPs are kept in check by MMP inhibitor TIMP-3, which directly binds and inactivates MMPs. The balance between TIMPs and MMPs is key for ECM stability, but, in CRC, MMP-2 and MMP-9 expression are increased, along with a downregulation of TIMP-3 and collagen type IV leading to degradation of ECM. These changes are favorable to cell proliferation, but, above all, also to invasion (Figure 1) [21].

As mentioned before, CAFs can facilitate invasion through the generation of tracks in the ECM by digesting the matrix and, thereby, allowing cancer cells to leave the site of origin (Figure 1) [5,22,23]. Force-mediated and protease-mediated matrix remodeling are required for the formation of these tracks. Particularly, Rho–ROCK and MMPs play a fundamental role for invasion of the cancer cells in this manner [24]. In accordance with this, Neri et al. demonstrated that podoplanin (PDPN)-expressing CAFs, can boost the invasion of cancer cells in lung adenocarcinoma through the activation of the Rho–ROCK pathway [25]. Inhibition of tracks could be a potential means of preventing invasion and, hence, metastasis.

### 2.2. CAFs Can Induce Stiffness of ECM

Matrix stiffness is defined as “the capacity of the matrix to resist deformation from an applied force” and is mainly dependent on ECM organization and composition [26]. Tumor ECM has been shown to be 1.5 times stiffer than normal tissues [27]. Stiffness promotes the assembly of actin-rich structures called invadosomes that are composed of invadopodia and podosomes, which are important for the migration of cells [28]. Maybe counterintuitively, multiple studies suggest that tumor stiffness is positively correlated with cancer invasion and metastasis (Figure 1) [26,28,29,30,31]. A study conducted on 337 breast cancer patients showed that higher matrix stiffness is associated with more aggressive cancer subtypes [32]. Enzymes produced by CAFs contribute to increased stiffness of tumor tissue and cause a higher synthesis of ECM as opposed to its degradation [5,33]. CAFs have the ability to digest the ECM in order to create tracks, as mentioned before, but they also increase its stiffness. These two apparently opposed processes give the idea of the complexity of these cells. Activated stromal cells produce LOXs, which are the primary driver of collagen cross-link formation [5,34,35]. Collagen cross-linking is able to promote matrix stiffness and cancer cell invasion [36]. When the YAP pathway is activated in CAFs, it promotes matrix stiffness, which, in turn, induces the activation of YAP in CAFs, enhancing a feedforward loop [37]. Torres et al. found lysyl oxidase like 2 (LOXL2) to be overexpressed in CAFs of CRC samples, proposing it as a predictive prognostic factor in colon cancer patients [38]. Therefore, targeting CAF-derived LOX oxidases has been suggested as a potential target against cancer migration and invasion [34].

## 3. The Role of Fibroblasts in EMT and Migration

Mesenchyme comes from a combination of the Greek words “mésos”, which means middle, and “enchyme”, which means cellular tissue. It refers to cells that develop into connective tissue, blood vessels, and lymphatic tissue. For the development of cancer cell metastasis, it is believed that cells need to undergo an additional step when the ECM is degraded: the acquisition of mesenchymal features. During tumor progression, the biological process of EMT occurs when epithelial cells acquire mesenchymal features (Figure 1).

EMT transcription factors (EMT-TF) are activated early in EMT and include TWIST, ZEB, and SNAIL/SLUG families. These markers can, in turn, upregulate a plethora of mesenchymal marker genes and repress those associated with an epithelial phenotype, such as genes involved in cell–cell adhesion and cell polarity [39]. It has been reported that SNAIL and ZEB play critical roles in EMT in CRC. SNAIL not only induces EMT, but also exerts cancer stem cell activities by activating interleukin-8 (IL-8) expression [40]. ZEB1 and ZEB2 are linked in a feedback loop with mir-200 and induce EMT and cancer progression [41]. STAT3 can bind directly to the ZEB1 promoter and induce its expression in CRC. In turn, ZEB1 downregulates E-cadherin, promoting EMT and invasion [42]. In CRC patients, increased ZEB2 levels strongly correlate with worse relapse-free survival and dismal prognosis [43,44]. Furthermore, Francescangeli et al. found that a population of quiescent/slow cycling cells are defined by ZEB2 expression and resist chemotherapy [43]. Two additional EMT transcription factors (TFs) appear to be implicated in CRC, i.e., forkhead box (FOX) family of TFs and the Prospero homeobox 1 (PROX1) transcription factor [45].

One of the key CAF-released inducers of the EMT-TFs, and most studied one, is transforming growth factor beta (TGF-β), which can modulate EMT via the TGF-βR/SMAD pathway [46,47]. TGF-β plays a dual role in cancers, as it can act in a tumor-suppressive manner in the early stages of tumor development, but can enhance tumor progression in later stages by promoting EMT and cell proliferation. In CRC, loss of SMAD4 has been shown to increase levels of TGF-β signaling and induce EMT [48]. Lamouille et al. demonstrated that TGF-β can activate mTORC2, an actor of the PI3K/AKT pathway, which can modulate the expression levels of genes relevant for the EMT process [49]. SMAD4 is also a negative regulator of STAT3 signaling. Knockdown of SMAD4 can, therefore, lead to aberrant STAT3 activation, which, in turn, can lead to EMT and expression of ZEB1 in CRC [45]. Overexpression of the WNT/β-catenin pathway is another EMT promoter in CRC invasion, in which both the canonical and noncanonical pathways are involved. IWR-1, a compound that stabilizes the β-catenin destruction complex, and thereby inhibits Wnt pathway activity, can inhibit EMT progression by suppressing survivin, a downstream WNT target gene [50]. An example of a receptor of the noncanonical WNT pathway involved in EMT is frizzled 2 (Fzd2). There is a high correlation between Fzd2, its ligands WNT5a/b, and EMT markers [51]. It has been shown that Fzd2 expression enhances EMT and cell migration via interaction with STAT3. Fzd2 has, therefore, been studied as a therapeutic target reducing metastases in xenograft mouse models of CRC [45]; however, further research is required to understand the role of Fzd2 and to develop it as a therapeutic target.

### 3.1. CAF-Secreted Factors 

Communication of tumor cells and fibroblasts via proinflammatory cytokines plays a crucial role in EMT. Some of these potentially metastatic CAF-secreted factors involved in EMT have been studied extensively in the past years in multiple cancer types, such as interleukin-6 (IL-6), osteopontin (OPN), hepatocyte growth factor (HGF), and CXCL12 [52,53,54]. CAF-derived IL-6 can induce EMT in multiple cancers, which is usually accompanied with an enhanced migratory capacity of cancer cells and consequential invasion [55,56,57]. We will discuss CAF-secreted IL-6 more in depth later on in this review. In cells undergoing EMT, there is usually an upregulation of several mesenchymal markers, such as vimentin, fibronectin, and N-cadherin, and a simultaneous downregulation of epithelial junction proteins, such as E-cadherin, occludins, and claudins. These regulations can cause destabilization of adherens junctions and, as a consequence, can facilitate migration and invasion.

In colorectal cancer, the cytokines HGF and OPN have been reported to be released by CAFs, causing EMT [46]. OPN is a key regulator of EMT through the TWIST pathway [58]. In addition, CXCL12 is reported to be a strong activator of EMT. It is suggested to activate the Wnt/β-catenin pathway via a CXCR4/CXCL12 axis, which leads to EMT and to therapy resistance. Normally, this interaction of CXCR4 with CXCL12 regulates stem cell trafficking [59,60], but hyperactivation of this axis commonly induces EMT in cancers [61]. CAFs are the biggest source of CXCL12 release in the tumor microenvironment (TME), which is further stimulated by TGF-β1 [61,62]. This CXCR4/CXCL12 axis has been reported to play an important role in CRC invasion and metastasis, and is being assessed as a therapeutic CRC target [63].

CAFs can also undergo lipidomic reprograming and secrete lipid metabolites that can be taken up by CRC cells and promote migration. This occurs partly through the overexpression of vimentin and the downregulation of E-cadherin [64]. This is a relatively new insight into the CRC invasion process and further research could provide a better understanding of CRC metastasis.

### 3.2. Stiffness in EMT

As mentioned before, ECM remodeling can result in a stiffer matrix, which can lead to tumor growth and metastasis. CAFs are key contributors to this ECM stiffness by tumor–CAF crosstalk or by inducing hypoxia within the tumor microenvironment [65]. Increased matrix rigidity has been shown to enhance the nuclear localization of transcription factor TWIST1 and its release from the cytoplasmic anchor G3BP2, promoting EMT [66]. Other well-known actors involved in EMT caused by stiffness are S100A11 membrane translocation, phosphorylation of elF4E, and autocrine TGF-β1. These converge on SNAIL expression, a well-known actor of EMT [67]. In accordance with this, an increased matrix rigidity induces EMT in pancreatic cancer [68]. In CRC, a stiffer matrix can increase the secretion of activin A from stromal cells, which, in turn, can induce invasion through the EMT-associated protein SNAIL. Inhibition of activin A may, therefore, represent an interesting approach to target the induction of EMT [30].

## 4. CAF–Tumor Communication

In ancient Greek mythology, Hermes was an Olympian deity and functioned as the messenger of gods. This ancient mythology can be applied to modern biomedical research: communication through messengers that can make the difference between ontogenesis and oncogenesis. One example is the multifunctional cytokine TGF-β that, in physiological conditions, regulates different mechanisms, such as morphogenesis, development, wound healing, and others, while, if deregulated, can promote aggressive phenotypes in cancers. Once activated, CAFs can secrete various messengers that can promote invasion and metastasis [69]. Some of these factors enhance EMT or ECM remodeling, as discussed in previous paragraphs. Here, we will focus on other mechanisms promoted by CAF-secreted factors.

### 4.1. Communication through Exosomes

Increasing evidence shows that exosomes are crucial in intercellular communication in cancer [70]. Exosomes are extracellular vesicles that are formed by invagination of the endosomal membrane, budding into multivesicular bodies (MVBs), and the subsequential release through fusion with the plasma membrane. Exosomes can be heterogeneous in size and contain a variety of substances, including microRNA (miRNA), messenger RNA (mRNA), DNA, proteins, and lipids. These exosome contents can act as paracrine and/or autocrine factors and can be both inside the exosomes, as well as exposed on the surface (such as TGF-β) [71]. Exosomes can be released by CAFs and internalized by cancer cells or the other way around, as cancer cells can release exosomes to change normal fibroblasts (NFs) into CAFs. Cancer-derived exosomes can induce differentiation of endothelial cells to CAFs, of which the exosomes, in turn, can aid in cancer cell invasion [72,73]. Recent research identified CAF-secreted exosomes as playing a critical role in tumor–CAF crosstalk and in cancer cell invasion [70]. CAF exosomes are enriched with TGF-β1, which induces the phosphorylation of SMAD2/3 in ovarian cancer cells, promoting EMT and invasion [70,74]. CAFs also secrete Wnt10b in exosomes, activating the Wnt/β-catenin pathway, EMT, and promoting breast cancer cell metastasis. In particular, fibroblasts that have low expression of p85α secrete more Wnt10b in exosomes, promoting, among others, migration and invasion of cancer cells [75].

Exosomes can also contain microRNAs (miRNAs), which are involved in the regulation of cancer. Among these miRNAs, miR-21 delivered by CAFs to CRC cells through exosomes has been shown to promote metastasis [76]. In breast cancer, the expression of CAF exosomal miR-21, miR-378e, and miR-143 promotes stemness and EMT [73]. In CRC, CAF-derived exosomes can contain miR-92a-3p, which promotes stemness, invasion, metastasis, chemotherapy resistance, and EMT. This miRNA targets FBXW7 and MOAP1, whose overexpression enhances mitochondrial apoptosis and inhibition of stemness, reverting migration, invasion, and therapeutic resistance [77]. Exosomal miR-17-5p secreted by CAFs targets RUNX3 in cancer cells, which enables MYC to activate the transcription of TGF-β1 to promote metastasis and, in turn, activate fibroblasts, forming a cancer positive feedback loop [78]. Recently, exosomes have been receiving increased attention as potential therapeutic targets in cancer research; however, for secretory pathways to be therapeutic targets, much more insight is needed to better understand the role of exosomes in tumor–CAF crosstalk.

### 4.2. Communication through TGF-β/HGF

As mentioned before, TGF-β and HGF play key roles in several processes of cancer development. TGF-β was initially discovered as a growth stimulant for rat fibroblasts, but was soon found to play a major role in tumor–CAF crosstalk. TGF-βs are cytokines that belong to the transforming growth factor superfamily. In cancer progression, it seems to play a dual role: initially, it is a suppressor of tumor progression, but then can turn into a promoter regulating fibroblast recruitment and activation [79]. It is widely accepted that TGF-β promotes the differentiation of fibroblasts into CAFs, which, in turn, secrete TGF-β, enhancing an autocrine signaling loop that maintains the activated status of fibroblasts [80,81]. Once activated, CAFs secrete high levels of TGF-β1 that can upregulate several EMT markers, such as vimentin, SNAIL, ZEB2, or long noncoding RNAs (lncRNAs) and downregulate E-cadherin [82,83,84]. CRC cells express TGF-β at an early phase and hyperactivate CAFs to express this cytokine. After mutational inactivation of TGF-β in CRC, CAFs become the new factories of TGF-β production [81].

The proposed role of TGF-β in CAF formation and induction of EMT suggests that targeting TGF-β could be an interesting approach against cancer invasion and metastasis [22]. In contrast, inhibition of TGF-β enhances expression of the pro-invasion factor HGF in CAFs, which suggests a negative regulation of TGF-β on HGF in fibroblasts [85]. According to this, TGF-β deficient fibroblasts increase the level of HGF, promoting invasion of mammary carcinoma cells, through MET tyrosine receptors [86].

HGF is mainly secreted by fibroblasts and is a growth factor that acts on epithelial cells. It interacts with MET to activate a signaling pathway that promotes cancer growth, survival, and invasion [87,88]. The constitutive activation of this pathway can occur through amplification or mutation in the MET gene in several tumors to evade regulatory mechanisms of cancer formation [89,90]. HGF can promote the transition from a preinvasive to an invasive phenotype of ductal carcinoma in situ (DCIS) cells. The activation of HGF/c-MET induces the degradation of collagen IV through the expression and secretion of the protease uPA and its receptor uPAR, which facilitates migration and invasion [91]. HGF secreted by CAFs can also upregulate the IL-6 receptor in gastric cancer cells. In turn, CAF-secreted IL-6 upregulates c-MET on the same cells. These two factors both collaborate to promote the activation of STAT3 and, consequently, of TWIST1, enhancing EMT and metastasis [92]. In the colon, HGF is secreted by smooth muscle cells lining the intestine, but appears preferentially active in the crypt base [93]. In CRC, as in many other cancers, overexpression of c-MET is correlated with poor prognosis and metastasis [93]. Stromal-secreted HGF has been shown to modulate WNT signaling and diminish GSK3 activity, which, in turn, stabilizes β-catenin. This induces cancer stem cell (CSC) features in tumor cells and enhances WNT signaling in tumor cells that reside close to CAFs [94]. Overall, TGF-β and HGF are two well-studied factors in CAF–tumor crosstalk and we will discuss the therapeutic implications further on in this review.

### 4.3. Communication through IL-6/IL-8

One of the most studied cytokines in cancer research is IL-6. It can be produced by many cells and it can promote tumor progression and therapeutic resistance [95]. IL-6 is involved in tumor–CAFs crosstalk, it can modulate the activation of fibroblasts, and, at the same time, support cancer cell growth. In particular, in esophageal carcinoma, binding its receptor (IL-6Rα), IL-6 can induce STAT3 and MEK/ERK signaling pathways, promoting proliferation and invasion of tumor cells [96]. As mentioned in the previous section, IL-6 has been associated with EMT and metastasis in different cancers. CAF-secreted IL-6 enhances migration and invasion of cancer cells, inducing the expression of EMT and metastatic-related genes. This process occurs through the activation of the JAK2/STAT3 pathway, both in lung cancer and gastric cancer [54,97]. In accordance with this, stromal IL-6 promotes EMT and, consequently, migration and therapeutic resistance in esophageal carcinoma [55]. It has been shown that CAF-derived IL-6 can also regulate the expression of osteopontin (OPN) in tumor cells, as OPN promotes invasion of head and neck cancer cells [98].

IL-8 is another molecule that plays a critical role in tumor progression and metastasis in different cancers. In ovarian cancers, CAF-derived IL-8 promotes normal fibroblast proliferation and stemness of tumor cells, activating the Notch-3 signaling pathway [99]. In addition, in pancreatic cancer, a subtype of CAFs with senescent features secretes more IL-8 than non-senescent CAFs. This senescent subset promotes pancreatic cancer cell invasion and metastasis [100]. Furthermore, melanoma cells cocultured with CAFs or treated with conditioned medium derived from CAFs display increased invasive potential. This can be reversed using IL-6 and IL-8 neutralizing antibodies, suggesting that the simultaneous inhibition of both cytokines is a promising approach in melanoma [101]. In CRC, IL-6 plays a significant clinical pathological role, as IL-6 is positively correlated with tumor TNM stage and associated with invasion depth and lymph node metastasis [102]. Furthermore, the expression of IL-6 and integrin *β*6 in CRC samples correlate with each other. IL-6 expression induces CRC invasion via the upregulation of integrin *β*6 through the IL-6 receptor/STAT-3 signaling [103]. Several clinical studies have been conducted targeting the IL-6 pathway in CRC; however, no significant anticancer effects have been observed yet with IL-6 monoclonal antibodies alone [104,105,106,107].

IL-8 is another potential therapeutic target, as the expression of IL-8 in the tumor microenvironment induces colon cancer growth and metastasis [108]. However, to date, the IL-8 inhibitors have also not yet been proven effective enough on their own [109,110,111].

### 4.4. Communication through Other Factors

Beside these major elements, there are many other factors that play a role in the induction of migration and invasion by CAFs. For example, interleukin-32 (IL-32) interacts with integrin-β3 on cancer cells and activates p38 MAPK pathway, which promotes EMT and invasion in breast cancer [112]. VCAM-1 is another highly secreted factor in CAF-conditioned medium as compared to conditioned medium of normal fibroblasts. This promotes growth and invasion of lung cancer cells through interaction with VLA-4 and the subsequent activation of the AKT and MAPK signaling pathway [113]. In CRC, CAFs secrete WNT2 in order to promote migration and invasion of cancer cells [114]. Furthermore, WNT2 acts in an autocrine manner in CRC by activating the canonical WNT signaling pathway in fibroblasts, and enhancing migration and invasion of both CAFs and CRC cells [115]. Moreover, CAFs stimulated with TGF-β secrete interleukin-11 (IL-11), which can promote metastasis in CRC through the GP130/STAT3 pathway [116].

## 5. CAF-Targeted Therapies

Therapy is derived quite literally from the Greek word “therapeia”, which means curing and healing. Differentially expressed CAF genes are potential targets for CAF-directed therapies. These genes are usually involved in carcinogenesis, angiogenesis, invasion, and metastasis. Targeting these genes and pathways opens possibilities to sensitize patients to therapies to reverse drug resistance or inhibit tumor progression. CAF targets are determined on genomic, transcriptional, and proteomic levels in CRC patients and cell lines. Many pathways are stimulated by CAFs, which offer a variety of available targets [5].

CAFs also have the ability to create an immunosuppressive TME, which inhibits both the innate and adaptive immune responses. CAFs can induce T cell anergy, inhibit T cell proliferation, recruit and activate T regulatory cells (Tregs), and prevent the access of immune cells to cancer cells through ECM remodeling [117]. In CRC, CAFs are able to recruit monocytes and facilitate their adhesion on tumor cells. Together with them, CAFs suppress the functions of natural killer cells (NK cells), which contribute to creating an immune-suppressive environment [118]. The ability of CAFs to promote the polarization of macrophages toward an immuno-suppressive and tumor-promoting phenotype has been described in different tumors [119,120]. CAFs can promote dysfunction and impair the cytotoxic function of NK cells. Similarly, in melanoma CAFs, secreting high levels of active MMPs decrease the lysing capacity of NK cells [121]. CAFs play a key role in cancer-related immune regulation and this topic deserves a more extensive review, for which we gladly refer to the overview by Ziani et al. [122].

As we will focus on signals that regulate EMT, migration, and invasion, we will not elucidate on immunotherapy. The therapies in the section are summarized in Table 1.

### 5.1. CAF Targeting via MMP9

MMPs are highly expressed in a wide range of tumor types and have been strongly implicated to play a key role in tumor invasion and metastasis [133]. An anti-MMP9 monoclonal antibody GS-5745 was shown to successfully inhibit tumor growth and metastasis in a colorectal cancer preclinical study in mice. However, in clinical trials, these MMP inhibitors did not show an antitumor effect [34,123]. Many clinical trials with MMP9 inhibitors have failed due to toxicity or insufficient clinical benefit. Toxicity was shown to be the consequence of MMP inhibitors circulating systemically; therefore, it would be interesting to focus on local application, such as targeted delivery or topical administration [134]. On top of that, failures were attributed to bad clinical design and non-specificity of MMP9 inhibitors [34]. As members of the MMP family are essential for the homeostasis maintenance, inflammatory responses, angiogenesis, and wound healing, it is of importance to focus on selectivity, as a broad range of MMP inhibitors can have detrimental consequences [135,136,137]. Furthermore, the specificity of these inhibitors is often low with off-target effects. However, a key reason for these failures might also be the lack of knowledge on MMP roles and functions in the ECM microenvironment. Therefore, MMP9 still remains a potential target that requires further research and optimization of specificity.

### 5.2. CAF Targeting via Hedgehog Signaling

Hedgehog (Hh) signaling is, in several cancers, a crucial modifier of CAFs and CAF-induced cancer growth, invasion, and metastasis [125,138,139,140]. Hedgehog signaling includes Sonic hedgehog (Shh), Desert hedgehog (Dhh), and Indian hedgehog (Ihh), all ligands that can bind to the transmembrane protein Patched [141]. In the absence of Hh, its ligand Patched represses the transmembrane receptor smoothened (SMO), leading to the proteolytic cleavage of full-length glioma-associated oncogene (Gli) to Gli repressor (GliR). Both Gli and GliR bind specific promoter regions and can, respectively, enhance or repress transcription of Hh target genes [141]. When bound by ligand, Patched no longer represses SMO and the Hh signaling pathway is activated, which has been shown to increase cell migration and invasion in pancreatic cancer and human gliomas by upregulating MMP9 expression [138]. As mutations in Patched1 or SMO can result in aberrant Hh pathway activation, SMO is often a target of the Hh pathway inhibitors in cancer therapeutics [142]. Inhibition of the Hh pathway or Shh ligands reportedly reduces tumor growth and distant metastases of PDAC [143]. Interestingly, inactivation of the Shh/Gli1 axis significantly reduces cell migration and invasiveness in breast cancer cells [125]. Desmoplasia in pancreatic cancer not only facilitates tumor growth, but is also suggested to protect tumor cells against chemotherapy due to elevated interstitial pressure, which prevents the chemotherapy from reaching the cancer cells. Here, the treatment with an SMO inhibitor decreased fibroblast numbers and increased vasculature density, leading to more effective chemotherapy delivery [124,143]. Unfortunately, clinical trials with PDAC patients using these SMO inhibitors in combination with gemcitabine have shown little to no efficacy [144]. Another phase II clinical trial of an SMO inhibitor and gemcitabine combination therapy was suspended when patients receiving this treatment had a worse clinical outcome than the group that received a placebo [143]. A proposed reason for these failures is that the desmoplastic reaction induced by Hh signaling also restrains pancreatic cancer cells from spreading to metastatic sites, which is abolished by the treatment. Balancing these effects of the stromal compartment may be crucial in the development of effective therapies.

### 5.3. CAF Targeting via TGF-β

TGF-β is a potential CAF-derived target that can potentially prevent metastasis by treating patients at an early stage. Different studies demonstrate that targeting this factor is a promising approach against CAF-mediated cancer progression [126,127,128,129,145].

The TGF-β receptor 1 inhibitor LY2109761 can affect tumor progression, targeting the crosstalk between stromal cells and cancer cells. In particular, this molecule can reduce the stromal component of hepatocellular carcinoma (HCC) and the production of connective tissue growth factor, which is highly produced by invasive HCC cells [126]. Galunisertib, another TGF-β receptor 1 inhibitor, prevents the TGF-β-induced fibroblast activation in ovarian cancer and, as a consequence, reduces migration and invasion of the cancer cells [127]. Similarly, in pancreatic cancer, inhibition of TGF-β activation of pancreatic stellate cells by the peptide hormone relaxin-2 can decrease tumor growth and potentiate the effect of the chemotherapeutic drug gemcitabine [128].

Artemisinin derivatives have also been shown to suppress TGF-β signaling in CAFs. This leads to the inactivation of CAFs and inhibition of cancer growth and metastasis. These results make artemisinin derivatives new potential therapeutic agents in breast cancer [129].

### 5.4. CAF Reprograming

The role of CAFs in promoting tumor progression prompted researchers to think that depletion of these activated stromal cells could be a potential therapeutic strategy. Surprisingly, the depletion of myofibroblasts in pancreatic ductal adenocarcinoma (PDAC) does not improve mice survival and leads to more aggressive, undifferentiated tumors [146,147]. Therefore, another approach for therapies against CAFs is the reprograming of tumor stroma. This approach consists of remodeling activated fibroblasts into quiescent fibroblasts that are normally present in homeostatic conditions. The aim of this approach is to inhibit the tumor-promoting functions of CAFs and CAF-induced therapy resistance. An example of this new strategy is in pancreatic cancer, where the hypovascularity of the stroma limits therapeutic efficacy. Based on the observation that quiescent pancreatic stellate cells (PSCs) store retinol, while the activated ones lose it, treatment of these cells with all-trans retinoic acid (ATRA) is assessed to restore physiological activity of PSCs. These reprogramed PSCs have the ability to produce secreted frizzled-related protein 4 (sFRP4), which reduces Wnt signaling in cancer cells and affects proliferation, apoptosis, and invasion of these cells [130]. A nano-system that combines ATRA and HSP47 small interfering RNA (siRNA), the latter of which is a collagen-specific chaperon crucial for the secretion of collagen protein into the ECM, was used to restore the physiological condition of PSCs. This reduces ECM production and, consequently, enhances pancreatic cancer chemotherapy [131]. In addition, vitamin D receptor (VDR) ligands promote the transition from activated to quiescent pancreatic stellate cells (PSCs), which lowers the secretion of tumor-promoting cytokines from these cells [132].

Currently, there are no main CAF-directed therapies used in CRC patients. Nevertheless, several of the inhibitory strategies described above are being tested. Applying such inhibitors, likely in combination with chemotherapy receptor-targeting therapies or radiotherapy, might improve the treatment of CRC patients and hopefully lower the incidence of metastatic spreading.

## 6. Conclusions

Cancer-related death is largely attributed to the complex metastatic process. The recognition that CAFs are central players in this process has boosted CAF research in the last decade. Understanding the underlying role CAFs play is, therefore, pivotal, as it seems that targeting CAFs may be necessary to prevent therapy resistance and metastasis. Clinical trials show promising results, but also prove that CAF-directed therapies are challenging and still have a long way to go to get from bench to bedside. Surprisingly, CAFs in CRC are still relatively understudied, despite the fact that CRC contains a subset with high levels of stromal cells [148]. The fact that this so-called CMS4 mesenchymal subtype has the worst prognosis, with an elevated metastatic capacity, makes it an interesting target for CAF-directed therapy. However, not much is known about the differences in the role of CAFs between the distinct CRC subtypes or even the specific role of CAFs in CMS4. Hence, further research is essential in this field to reduce cancer-related deaths due to metastasis. Research on this topic has shown that targeting CAFs can have opposing outcomes and sometimes result in an even more aggressive phenotype. Therefore, it is of importance to get a better understanding of different populations of fibroblasts surrounding tumors and specifically target those causing invasion and metastasis.

## Figures and Tables

**Table 1:** Summary of CAF-targeted therapies.

Pathway	Compound	Targets	Cancer	Mechanism	Ref.
MMP9	GS-5745	MMP9	Colorectal	Inhibition of tumor growth and metastasis	[123]
Hedgehog		Smo	Pancreatic	Decrease in fibroblast accumulation and easier drug delivery	[124]
		Shh/Gli pathway	Breast	Reduction in cell migration and invasion	[125]
TGF-β	LY2109761	TGF-β receptor1	Hepatic	Reduction in the stromal compartment	[126]
	Galunisertib		Ovarian	Prevention of CAF activation and reduction in proliferation and invasion	[127]
	Realaxin-2	Smad2 pathway	Pancreatic	Decrease in tumor growth and increase in sensitivity of gemcitabine	[128]
	Artesiminin	TGF-β	Breast	Inactivation of CAFs and inhibition of cancer growth and metastasis	[129]
Reprograming	ATRA	HSP47	Pancreatic	Reprograming of PSCs, reduction in Wnt signaling, and modulation of proliferation, apoptosis, and invasion	[130]
	Nano-system (ATRA+ anti HSP47)			Reprograming of PSCs, reduction in ECM production, and increase in chemotherapeutic effects	[131]
	VDR ligand			Induction of quiescent PSCs from activated PSCs	[132]
